# Antithrombotic Therapy Following Structural Heart Disease Interventions: Current Status and Future Directions

**DOI:** 10.31083/j.rcm2502060

**Published:** 2024-02-05

**Authors:** Andreas Mitsis, Michaela Kyriakou, Evi Christodoulou, Stefanos Sakellaropoulos, Panayiotis Avraamides

**Affiliations:** ^1^Cardiology Department, Nicosia General Hospital, 2029 Nicosia, Cyprus; ^2^Cardiology Department, Limassol General Hospital, 3304 Nicosia, Cyprus; ^3^Department of Internal Medicine, Cardiology Clinic, Kantonsspital Baden, 5404 Baden, Switzerland

**Keywords:** antiplatelets, antithrombotics, ASD, atrial appendage, atrial fibrillation, LAA, mitral valve, TAVI, transcatheter aortic valve implantation, TEER, TMVR, patent foramen ovale

## Abstract

Interventions in structural heart disease cover many catheter-based procedures for congenital and acquired conditions including valvular diseases, septal defects, arterial or venous obstructions, and fistulas. Among the available procedures, the most common are aortic valve implantation, mitral or tricuspid valve repair/implantation, left atrial appendage occlusion, and patent foramen ovale closure. Antithrombotic therapy for transcatheter structural heart disease interventions aims to prevent thromboembolic events and reduce the risk of short-term and long-term complications. The specific approach to antithrombotic therapy depends on the type of intervention and individual patient factors. In this review, we synopsize contemporary evidence on antithrombotic therapies for structural heart disease interventions and highlight the importance of a personalized approach. These recommendations may evolve over time as new evidence emerges and clinical guidelines are updated. Therefore, it’s crucial for healthcare professionals to stay updated on the most recent guidelines and individualize therapy based on patient-specific factors and procedural considerations.

## 1. Introduction

Catheter-based interventional cardiology procedures for structural heart disease have experienced remarkable evolution in recent decades, transforming the management of complex cardiovascular diseases. Nowadays these interventions are considered first-line treatment methods, providing alternatives to traditional open-heart surgeries, and enabling quicker recovery for patients.

Catheter based interventions have been progressed for the management of valvular conditions, including transcatheter aortic valve implantation (TAVI) for the management of severe aortic stenosis (SAS), transcatheter edge to edge repair (TEER) for the management of severe mitral (MR) and tricuspid regurgitation (TR). Catheter-based interventions have also been developed for other structural heart conditions. Closure devices are used to seal patent foramen ovale (PFO), atrial septal defects (ASDs) or ventricular septal defects (VSDs). Additionally, left atrial appendage (LAA) occlusion procedures have been developed to reduce the risk of stroke in patients with atrial fibrillation (AF) who are unable to tolerate or to whom long-term anticoagulation is contraindicated [1].

The evolution of catheter-based interventional cardiology procedures for structural heart disease has been driven by advancements in technology, imaging modalities, and procedural techniques. Following a structural heart disease intervention, the necessity of antithrombotic therapy is determined based on several factors, including the type of intervention, individual patient characteristics, and the presence of other indications for anticoagulation or antiplatelet therapy. The primary goals of antithrombotic therapy in these cases are to prevent thrombus formation, minimize the risk of embolism or thrombus-related complications, and ensure optimal long-term outcomes. Mostly, antiplatelets [Aspirin (ASA) and/or Clopidogrel], indirect anticoagulants (e.g., Vitamin K antagonists) or direct oral anticoagulants (DOACs) are used.

The purpose of this article is to review the current evidence on antithrombotic therapies for structural heart disease interventions and highlight the importance of a personalized approach in each patient.

## 2. Discussion

### 2.1 Mechanism of Device Thrombosis

**Fig. 1.**
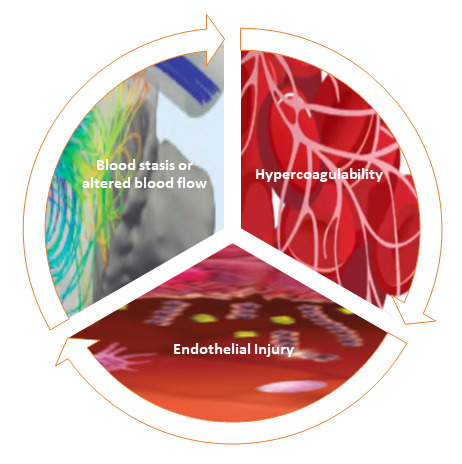
**Illustration of virchow’s triad in device-related thrombosis**. The figure depicts the three key factors—endothelial injury, altered blood flow, and hypercoagulability—comprising Virchow’s Triad, contributing to thrombosis formation on the device surface.

Implantable cardiac devices for the treatment of structural heart disease are made of various materials, and their introduction into the cardiovascular system can trigger a cascade of events that may lead to thrombus formation. The mechanisms of device thrombosis in cardiac structural heart disease interventions can be multifactorial and include several factors but this process always follows the classical concept of the Virchow’s Triad; endothelial injury, stasis or altered blood flow, hypercoagulability [2] (Fig. 1).

During cardiac interventions, the introduction and deployment of devices can cause endothelial injury. The disruption of the normal vascular architecture triggers a cascade of events, promoting local inflammatory response and leading to platelet adhesion and activation, providing a substrate for thrombus formation [3].

**Fig. 2.**
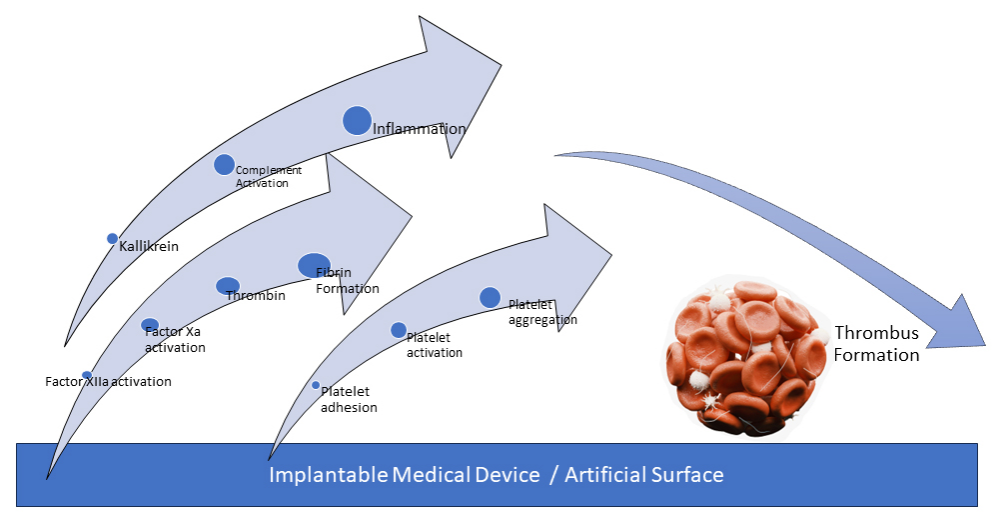
**Schematic representation of contact activation on artificial surface leading to device thrombosis**. The figure illustrates the stepwise process by which contact activation occurs on the artificial surface, ultimately resulting in thrombosis of the device.

Moreover, implantable cardiac devices have a prothrombotic surface that has the potential to trigger the activation of the coagulation system through intricate interactions between blood cells and plasma proteins [3]. The adsorption of proteins onto the surface of medical devices prompts platelet adhesion, activation, and aggregation [4]. When Factor XII adheres to the surface, it undergoes autoactivation, leading to the conversion of prekallikrein to kallikrein and initiating the processes of coagulation and thrombin generation. Beyond facilitating fibrin deposition on the surface, thrombin plays a role in enhancing platelet activation. The aggregates of platelets deposited on the surface are further stabilized by fibrin strands, forming a cohesive platelet–fibrin thrombus [4]. Notably, kallikrein, thrombin, and other coagulation enzymes activate complement, thereby inducing a localized inflammatory response (Fig. 2).

Finally, changes in blood flow patterns, and shear stress in the vicinity of the implanted device such as turbulence or stagnation, can promote thrombosis. The introduction of devices can alter the normal hemodynamic of blood flow, creating areas of stasis or disturbed flow that enhance the risk of clot formation. This is particularly relevant in areas where the devices are implanted, and blood flow may become turbulent. Besides, improper sizing, mal-positioning, or incomplete expansion of the device can create areas where blood flow is disturbed, increasing the risk of thrombosis [5].

### 2.2 Transcatheter Aortic Valve Implantation 

TAVI has revolutionized the treatment of SAS and has expanded treatment options to patients who are at high surgical risk or deemed inoperable to even intermediate and lower risk patients [6, 7]. Despite this advancement, the challenges posed by ischemic and embolic post-procedural related complications, as well as hemorrhagic events, continue to be crucial factors and associated with mortality. Within this context, the most effective antithrombotic regimen following a successful TAVI lacks clarity. Despite several randomized trials (Table 1) many recommendations are still based on expert opinion.

**Table 1.** **Summary of Randomized Trials Investigating Antithrombotic Therapy Following Transcatheter Aortic Valve Implantation, in patients with or without indication to long-term OAC**.

Study	Year	Participants	Patients characteristics	Antithrombotic therapy	Clinical outcome
Ussia *et al*.	2011	79	Patients without indication to long-term OAC	3 months DAPT followed by ASA alone vs. ASA alone	No difference between DAPT vs. ASA at 30 days and 6 months
SAT-TAVI	2014	120	Patients without indication to long-term OAC	6 months DAPT vs. ASA alone	No difference in the VARC combined safety end point at 30 days, no differences in the clinical status at 6 months
ARTE	2017	222	Patients without indication to long-term OAC	3 months DAPT vs. ASA alone	In the DAPT group, there was a trend towards a higher incidence of the composite outcome or major or life-threatening bleeding
POPULAR TAVI (Cohort A)	2020	665	Patients without indication to long-term OAC	ASA alone vs. 3 months DAPT followed by ASA alone	ASA monotherapy was associated with a reduction in the occurrence of bleeding events
GALILEO	2020	1644	Patients without indication to long-term OAC	Rivaroxaban 10 mg/d (with ASA for the first 3 months) vs. ASA (with Clopidogrel 75 mg/d for the first 3 months)	Rivaroxaban group exhibited a higher incidence of the composite outcome of death or the first thromboembolic event and numerical increase of bleeding events
POPULAR TAVI (Cohort B)	2020	313	Patients with indication to long-term OAC	OAC alone vs. OAC with Clopidogrel for the first 3 months followed by OAC alone	OAC monotherapy was associated with reduced incidence of bleeding events, without a simultaneous increase in thrombotic events
ATLANTIS (1st Stratum)	2022	451	Patients with indication to long-term OAC	Apixaban vs. vitamin K antagonists	Apixaban was associated with a higher risk for the composite of death, any stroke or transient ischemic attack
ATLANTIS (2nd Stratum)	2022	1049	Patients without indication to long-term OAC	Apixaban vs. antiplatelet therapy (ASA or DAPT)	The hazard ratio for apixaban versus antiplatelet therapy (single or dual) was 0.88 (95% CI: 0.66–1.17)

ASA, aspirin; DAPT, dual antiplatelet therapy (ASA 75–100 mg/day plus Clopidogrel 75 mg/day); SAT-TAVI, single antiplatelet therapy for transcatheter aortic valve implantation; OAC, oral anticoagulation; VARC, valve academic research consortium; 95% CI, 95% confidence interval.

Most of the thrombotic events post TAVI occurs during the first 48–72 h after valve implantation and are likely related to acute embolization of fibro-calcific valve material or catheter manipulation damaging aortic wall. Later ischemic events may be linked to thrombosis of the prosthesis surface or to unrecognized/new onset of atrial fibrillation [8]. Of note, anatomopathological analyses support the 3 months antithrombotic strategy. A recent study showed that neointimal tissue infiltration and full endothelialisation of the valve stent frame occur approximately 3 months after the procedure, with a decrease in thromboembolic events thereafter [9].

The dedicated antithrombotic therapy post-TAVI has been evolved over the years. During early days of TAVI, a common approach was to initiate dual antiplatelet therapy (DAPT) with aspirin and clopidogrel for a limited duration following the intervention. The duration of DAPT typically ranged from 3 to 6 months, although it could be extended in certain cases based on individual patient characteristics and procedural factors. Many studies have been designed to test this DAPT antithrombotic strategy. Ussia *et al*. [10], showed that the strategy of adding clopidogrel to aspirin for 3 months after TAVI was not found to be superior to single antiplatelet therapy (SAPT) with aspirin alone. Similarly, SAT-TAVI [11] and ARTE [12] studies showed that TAVI procedures can be performed without DAPT, without any cost in the morbidity and mortality.

The SAT-TAVI (Single Antiplatelet Therapy for Transcatheter Aortic Valve Implantation) trial involved 120 patients undergoing TAVI, randomly assigned to either the DAPT group, receiving aspirin and clopidogrel 75 mg/od or ticlopidine 500 mg/bid, or the Aspirin-Only (ASA) group. No significant disparities were observed in the Valve Academic Research Consortium (VARC) combined 30-day safety endpoint, all-cause mortality, and cardiovascular mortality between the two groups. However, at the 30-day mark, the ASA group exhibited a noteworthy reduction in vascular complications (*p <* 0.05). No discernible distinctions in clinical status were noted between the groups up to the 6-month follow-up period [11].

The ARTE (Aspirin Versus Aspirin + Clopidogrel Following Transcatheter Aortic Valve Implantation) trial, a study involving 222 patients, aimed to compare the outcomes of aspirin plus clopidogrel versus aspirin alone following the TAVI procedure in patients receiving balloon-expandable valves. The composite of death, myocardial infarction (MI), stroke, transient ischemic attack, or major or life-threatening bleeding showed a tendency to occur more frequently in the DAPT group (15.3% vs. 7.2%, *p =* 0.065). However, there were no significant differences between the groups in terms of death (DAPT, 6.3%; Single Antiplatelet Therapy (SAPT), 3.6%; *p =* 0.37), MI (DAPT, 3.6%; SAPT, 0.9%; *p =* 0.18), or stroke/transient ischemic attack (DAPT, 2.7%; SAPT, 0.9%; *p =* 0.31) at 3 months. The DAPT group exhibited a higher incidence of major or life-threatening bleeding events (10.8% vs. 3.6% in the SAPT group, *p =* 0.038). Single antiplatelet therapy was associated with a reduced risk of major or life-threatening bleeding events without an increased risk of MI or stroke [12].

The POPULAR TAVI (Antiplatelet Therapy for Patients Undergoing Transcatheter Aortic Valve Implantation) trial validated the safety of monotherapy compared to DAPT following TAVI. The occurrence of bleeding and the combined incidence of bleeding or thromboembolic events at the one-year mark were notably less frequent with aspirin alone than with the combination of aspirin and clopidogrel administered over a three-month period [13]. In the cohort A of the trial, 665 patients without an indication for long-term oral anticoagulation (OAC) were randomly assigned in an open-label manner, to receive either aspirin alone at a dose of 80–100 mg per day or DAPT with aspirin and clopidogrel for a duration of 3 months, followed by aspirin alone. The study findings revealed that aspirin monotherapy was associated with a notable reduction in the occurrence of bleeding events, including major, life-threatening, or disabling bleeding incidents (*p* = 0.001). While aspirin alone demonstrated noninferiority compared to the combination of aspirin and clopidogrel in terms of the composite outcome, which encompassed thromboembolic events such as cardiovascular-related mortality, ischemic stroke, or myocardial infarction, it did not exhibit superiority in this regard [13].

Furthermore, the exploration of dual therapy involving a direct oral anticoagulant (DOAC) in TAVI patients who did not require oral anticoagulation therapy was initially undertaken in the GALILEO trial [14]. GALILEO (Global Study Comparing a Rivaroxaban-based Antithrombotic Strategy to an Antiplatelet-based Strategy after Transcatheter Aortic Valve Replacement to Optimize Clinical Outcomes) trial randomized 1644 patients into two groups: one receiving dual therapy (comprising rivaroxaban 10 mg daily and aspirin 75–100 mg daily for the initial 3 months) and the other receiving aspirin alone at a daily dose of 75 to 100 mg (along with clopidogrel 75 mg daily for the first 3 months). Of note, the trial was prematurely halted due to safety concerns observed in the dual therapy group. After a median follow-up duration of 17 months, patients in the dual therapy group exhibited a higher incidence of the composite outcome of death or the first thromboembolic event (*p =* 0.04). Additionally, there was a numerical increase in major, disabling, or life-threatening bleeding events (*p =*0.08) [14].

The ATLANTIS (Anti-Thrombotic Strategy to Lower All Cardiovascular and Neurologic Ischemic and Hemorrhagic Events after Trans-Aortic Valve Implantation for Aortic Stenosis trial) – Stratum 2 specifically structured to establish the superiority of apixaban over the standard of care (vitamin K antagonists for patients with an established indication for OAC or antiplatelet therapy for patients without indication for OAC) after TAVI [15]. The trial aimed to assess the effectiveness and safety of a 5 mg twice-daily dose of apixaban when compared to the established standard of care, which involved SAPT/DAPT in patients without indication for OAC. The trial’s findings indicate that apixaban does not exhibit superiority over standard of care. The primary endpoint, which includes a composite of death, stroke, myocardial infarction, systemic emboli, intracardiac or valve thrombosis, and deep vein thrombosis/pulmonary embolism, showed a similar occurrence for apixaban compared to standard of care, a finding that remained consistent when valve thrombosis was excluded [15].

Also, in accordance with a recent systemic meta-analysis, at the 30-day post- TAVI mark, there were no discernible differences in outcomes such as all-cause mortality (7.3% vs. 6%, *p = *0.57), cardiovascular mortality (5% vs. 6%, *p = *0.76), stroke (*p = *0.57), and myocardial infarction (*p = *0.59) between patients receiving DAPT and those receiving SAPT. However, it’s noteworthy that individuals in the DAPT group exhibited a notably elevated incidence of severe and major bleeding events during this 30-day follow-up period (18% vs. 7%, *p = *0.004) [16].

At present, both American and European guidelines, support the use of single antiplatelet therapy after the intervention [17, 18], unless there is another reason for DAPT due to an elevated ischemic risk (e.g., recent acute coronary syndrome, coronary stent implantation, coronary artery bypass grafting, peripheral artery revascularization, or stroke) (Table 2) [19]. After completion of DAPT, the guidelines suggest continuing with SAPT (aspirin or clopidogrel) for up to 6 months to 1 year. The choice between aspirin and clopidogrel depends on individual patient factors, such as bleeding risk and concomitant indications.

**Table 2.** **Summary of evidence regarding antithrombotic therapy following Transcatheter Aortic Valve Implantation**.

Antithrombotic therapy after Transcatheter Aortic Valve Implantation			
No Indication for long-term anticoagulation		Indication for anticoagulation	
Low ischaemic risk and/or high bleeding risk patients	High ischaemic risk and/or low bleeding risk patients	High ischaemic risk and/or low bleeding risk patients	Low ischaemic risk and/or high bleeding risk patients
SAPT (ASA or Clopidogrel) lifelong	DAPT for 1–6 months followed by SAPT (ASA or Clopidogrel) lifelong	OAC and SAPT (preferable Clopidogrel) for 1–6 months and then OAC lifelong	OAC monotherapy lifelong

Ischemic risk is considered elevated after an acute coronary syndrome, implantation of coronary stent, coronary artery bypass, peripheral artery disease, or stroke. Bleeding risk is considered elevated in elderly patients, in frailty, after history of GIH, elevated HAS-BLED score, anemia, thrombocytopenia, renal failure, hemorrhagic stroke etc. ASA, aspirin; DAPT, dual antiplatelet therapy; GIH, gastro-intestinal hemorrhage; OAC, oral anticoagulation therapy; SAPT, single antiplatelet therapy.

For patients with additional indications for OAC (e.g., AF, mechanical heart valve), the decision to use anticoagulation along with antiplatelet therapy should be individualized based on the balance between thrombotic and bleeding risks. Taking under consideration that these patients are usually old and frail, with an elevated bleeding risk, monotherapy with OACs seems reasonable, unless in a coexistent elevated ischemic risk where dual therapy with OAC and SAPT for a period 1–6 moths seems reasonable (Table 2).

Previous observational studies have undertaken assessments, comparing outcomes between those managed with OAC alone and those subjected to a regimen combining OAC with antiplatelet therapy. The findings from these studies notably support the use of OACs as a standalone strategy, given its safety profile characterized by lower rates of bleeding events. Furthermore, OAC monotherapy is demonstrated to be noninferior when compared to the combination of OACs and clopidogrel concerning key clinical endpoints, including overall mortality, cardiovascular mortality, and ischemic events.

In the POPULAR-TAVI trial’s cohort B, a total of 313 TAVI patients who required long-term OAC therapy, were randomly divided into two groups: one receiving OAC alone and the other receiving OAC in combination with a three-month course of clopidogrel. The trial’s findings led to the conclusion that the administration of OAC in isolation resulted in a diminished occurrence of bleeding events, and notably, it did so without concomitantly elevating the incidence of thrombotic events or cardiovascular mortality [13].

The ATLANTIS trial, specifically designed within Stratum 1 [15], was structured with the aim of establishing whether apixaban could surpass the conventional standard of care, particularly Vitamin K Antagonists (VKA), in patients requiring OAC. Nevertheless, the trial’s outcomes reveal that apixaban does not demonstrate superiority over VKA. This conclusion is supported by the hazard ratio for the primary outcome, which was 1.02, and for the primary safety endpoint, which was 0.91. However, it’s important to note that non-inferiority was demonstrated in the trial results.

The question surrounding the potential replacement VKAs with DOACs in patients undergoing TAVI remains a subject of ongoing debate. DOACs have gained broad acceptance in patients with nonvalvular AF, as they have shown noninferiority to VKA in preventing thromboembolic events with specific agents like dabigatran, rivaroxaban, and edoxaban [20]. A study conducted by Tanawuttiwat *et al*. [21], including a cohort of 21,131 patients with indications for OAC, drawn from the STS/ACC TVT (Society of Thoracic Surgeons/American College of Cardiology Transcatheter valve therapy) Registry, revealed no significant difference in one-year stroke rates (2.51% vs. 2.37% for DOAC and VKA, respectively; *p =* 0.980). However, it showed a lower rate of one-year bleeding events, intracranial hemorrhage, and mortality associated with DOACs in comparison to VKA [21].

In addition, in the combined France-TAVI and France-2 registries, a total of 8962 patients received OAC therapy following TAVI with 2180 (24%) of them prescribed DOACs and 6782 (76%) VKAs. After a three-year follow-up and propensity score matching, the data revealed an increase in mortality rates associated with VKAs compared to DOACs (VKA vs. DOAC: 35.6% vs. 31.2%; *p* < 0.005), as well as in major bleeding events (VKA 12.3% vs. DOAC 8.4%; *p* < 0.005). However, no notable differences were observed between the two groups concerning the occurrence of ischemic stroke and acute coronary syndrome [22].

Moreover, a recent comprehensive meta-analysis was conducted involving 30,388 patients who underwent TAVI and had AF, with the aim to assess the comparative efficacy of DOACs with VKAs. The analysis did not reveal a statistically significant difference in stroke incidence between the DOACs group and the VKAs group. However, it’s worth noting that the DOACs group displayed a numerically higher but non-significant number of composite endpoint events when compared to the VKAs group. Nevertheless, the incidence of major bleeding events was lower in the DOACs group (11.29% vs. 13.89%, *p* < 0.00001), as was the rate of all-cause mortality (14.18% vs. 17.61%, *p <* 0.00001) compared to the VKAs group. In summary, these findings suggest that the adoption of DOACs is associated with a diminished incidence of major bleeding and decreased all-cause mortality [23].

Consequently, clinicians should consider the unique characteristics of each patient and assess personalized bleeding risk when deliberating on the optimal anticoagulation regimen. This individualized approach is essential for the optimization of patient outcomes in the post-TAVI setting (Table 2).

### 2.3 Transcatheter Edge-to-Edge Repair of Mitral Valve

TEER has become an important tool the management of severe symptomatic MR and TR in patient without surgical option. Based on the surgical Alfieri technique, TEER technique uses a clipping device that grasps the valve leaflets thereby creating a “double orifice” valve area [24]. At present, there are two commercially available devices with Conformité Européene (CE) Mark; Abbott offers the MitralClip and the TriClip system for the mitral and tricuspid valve respectively. Edwards developed the Pascal devices to treat both valves. The Endovascular Valve Edge-to-Edge Repair Study (EVEREST) compared TEER with the MitraClip device to conventional surgery for primary mitral regurgitation, enrolling 279 patients with grade 3+ or 4+ MR, with outcomes demonstrating efficacy and safety at 12 months [25] and 5-year follow up [26]. The COAPT trial enrolled patients with symptomatic heart failure and moderate-to-severe or severe secondary MR, showing that TEER with the MitraClip device, in addition to medical therapy, significantly reduced heart failure hospitalizations and overall mortality compared to medical therapy alone [27].

Given the absence of specific guidelines, the choice of antithrombotic therapy after TEER is based on the design of these landmark trials and individualized on patient characteristics (thromboembolic vs. bleeding risk), procedural factors, and the presence of other indications for anticoagulation or antiplatelet therapy (Table 3).

**Table 3.** **Summary of evidence regarding antithrombotic therapy following Transcatheter Mitral or Tricuspid Valve Interventions**.

Antithrombotic therapy after Transcatheter Mitral or Tricuspid Valve Interventions			
Transcatheter Edge to Edge Repair		Transcatheter Valve Replacement	
Concomitant AF	No Indication for long-term anticoagulation	Low thrombotic risk and/or high bleeding risk	High thrombotic risk and/or low bleeding risk
OAC with VKA with target INR 2.5–3 lifelong	DAPT for 1–6 months and then ASA lifelong	OAC with VKA and target INR 2.5–3 for 3 months	OAC with VKA and target INR 2.5–3 for 6 months
		Then continue with ASA lifelong unless other reason for OAC (e.g., AF)	

Thrombotic risk can be elevated due to patient characteristics (e.g., increased age, left ventricular dysfunction, hypercoagulable state) or procedural related factors (tricuspid site procedure, valve in valve procedures, type of device). Bleeding risk is considered elevated in elderly patients, in frailty, after history of GIH, elevated HAS-BLED score, anemia, thrombocytopenia, renal failure, hemorrhagic stroke etc. AF, atrial fibrillation; DAPT, dual antiplatelet therapy; ASA, aspirin; INR, international normalized ratio; GIH, gastro-intestinal hemorrhage; OAC, oral anticoagulation therapy; VKA, vitamin-K antagonists.

In general, after a TEER procedure, a common approach is to use DAPT with aspirin and clopidogrel for a limited duration. The duration of DAPT may vary, but it is often continued for several months, like the recommendations for other transcatheter interventions. In the EVEREST I trial [28], EVEREST II study protocol [29], EVEREST II RCT [25] and the EVEREST II high risk registry (HRR) [30], a regimen of aspirin at a dose of 325 mg daily for 6 months to 1 year was used associated with clopidogrel at a dose of 75 mg daily for 1 month. In the Cardiovascular Outcomes Assessment of the MitraClip Percutaneous Therapy for Heart Failure Patients with Functional Mitral Regurgitation (COAPT) trial, standard regimen included aspirin, 81 mg/day, and/or clopidogrel, 75 mg/day, was used for 6 months or longer [27].

Current practice is to recommend DAPT with ASA and Clopidogrel for a period of 3 to 6 months, depending on the individualized bleeding risk of each patient, and then to continue with ASA lifelong. Of note, these recommendations have not been evaluated in controlled randomized trials.

AF is not uncommon comorbidity in patients with moderate or severe MR as showed in the large registries Real World Expanded Multi-center Study of the MitraClip System (REALISM) [31] and A Two-Phase Observational Study of the MitraClip System in Europe (ACCESS-EU) [32] which report coexisting AF in 66.5% and 67.7% of TEER patients, respectively. A recent multicenter, observation study, showed that the prevalence of concomitant AF in patients who underwent TEER was more than 75% and the majority of patients received postprocedural antithrombotic therapy consisting of an oral anticoagulant [33]. Overall, VKAs were used most frequently compared with DOACs (52.1% vs. 47.9%, respectively). Post-procedurally, in patients with indication for OAC, the combination of OAC + SAPT was used most frequently (55.2%), followed by OAC monotherapy (32.6%) and OAC + DAPT combination (12.2%). The remaining patients without an indication for OAC (26.3%) received ASA pre-procedurally (88.7%) and were predominantly switched to DAPT with the addition of clopidogrel after TEER (82.5%) [33]. Current practice for patients with AF and a clear indication for lifelong OAC is to maintain OAC with VKA and a target international normalized ratio (INR) 2.5.

### 2.4 Transcatheter Mitral Valve Replacement

Transcatheter mitral valve replacement (TMVR) represent a new therapeutic opportunity for patients with mitral valve disease and no option for surgical mitral valve replacement(MVR) or TEER. The last years, new dedicated devices have been presented [34], while new indications like the treatment of degenerated bio-prostheses (valve-in-valve [ViV]), failed annuloplasty rings (valve-in-ring [ViR]), and severe mitral annular calcification (valve-in-mitral annular calcification [ViMAC]) have been also appeared [35].

TMVR is still an evolving field, and specific guidelines for antithrombotic therapy after TMVR have not been established. Patients treated with TMVR are exposed to an increased risk of valve thrombosis and thromboembolic event. In clinical practice the most common approach is to follow the current recommendation for surgical bioprosthetic MVR. After surgical bioprosthetic MVR, current guidelines support the use of OAC with VKA and a target INR 2.5 for 3–6 months, as it is known that endothelialisation is usually complete after 90 days after the implantation of the valve [36]. Prolongation of the OAC for more than 6 months after aortic valve replacement (AVR) has showed to be related with improved survival and less thromboembolic events but more bleeding episodes [37]. Taking under consideration that mitral site is more thrombogenic than the aortic site due to lower local blood flow perturbations around the valve prosthesis, oral anticoagulation with vitamin K antagonist (VKA) seems reasonable to be maintained for at least 6 months [36].

TMVR in native mitral valve has been shown to have increase thrombogenicity as a procedure. In the feasibility trial of the Tendyne valve (Abbot), 6 cases of thrombosis were reported at 1-year follow-up (6 of 100 patients, rate 6.0%), all observed in the early part of the study (first 35 cases), when post-operative medical therapy comprised only of aspirin. After these thrombotic episodes, the study protocol changed applying the use of mandatory VKA therapy (target INR 2.5 to 3.5) for at least 3 months, and no further cases of valve thrombosis were observed [38]. In contrast, no cases of clinically overt valve thrombosis at 1 year were reported after Intrepid valve (Medtronic) procedure because of the antithrombotic strategy with VKA (target INR 2.5 to 3.5) plus SAPT for at least 3 months. Of note, this combination had as a result a relatively high rate (18%) of 30-day major bleeding [39]. Taking under consideration all the above, OAC with VKA and a target INR around 2.5 for 3-6 months should be considered after TMVR [40]. Oral anticoagulation is recommended lifelong for patients who have other indications for anticoagulation, like AF.

TMVR has found application in cases involving a degenerated mitral valve surgical bio-prosthesis (ViV), unsuccessful mitral valve repairs with an annuloplasty ring (valve-in-ring), and significant mitral annular calcification (valve-in-mitral annular calcification). In such scenarios, the utilization of the Edwards Sapien Valve has emerged as a viable therapeutic choice for managing degenerated bioprosthetic valves and annuloplasty rings that have failed, particularly in patients considered to be at high or prohibitive surgical risk [35]. Information gleaned from the largest multicenter TMVR registry revealed instances of valve thrombosis in 10 cases (4.2%), occurring at various intervals ranging from the initial days to up to 2 years following TMVR. Notably, 71.8% of the patients in the study received anticoagulant therapy post-TMVR, while the remaining 28.2% were administered antiplatelet therapy. Intriguingly, the cumulative one-year incidence of valve thrombosis was markedly higher in patients who did not receive anticoagulation in comparison to those who did (6.6% vs. 1.6%; *p =* 0.019) [35]. Similarly, a single center TMVR registry, showed that a 2-year rate of re-intervention and valve thrombosis were 8.8% and 14.4%, respectively [41].

Taking under consideration all the above, it seems reasonable to prescribe oral anticoagulation with VKA the first months after any TMVR procedure in patients who do have not an indication for long-term anticoagulation, to minimize the risk of valve thrombosis. Further personalized treatment may vary based on the specific patient characteristics, procedural considerations, and the presence of other indications for anticoagulation or antiplatelet therapy (Table 3).

### 2.5 Transcatheter Edge to Edge Repair of Tricuspid Valve

Tricuspid regurgitation (TR) is frequently observed in individuals with left-sided valvular or myocardial conditions, often indicating an advanced stage of chronic heart failure with an unfavorable prognosis [42]. Even in the present day, isolated tricuspid valve surgery remains uncommon and is associated with the highest mortality rate among all types of valve procedures [43]. Therefore, in recent times, a great evolution of multiple percutaneous therapies and mainly TR-TEER, has been developed for treating severe tricuspid regurgitation. Other therapeutic options include procedures for annuloplasty (i.e., Cardioband), and finally, dedicated native tricuspid valve orthotopic valve implantation (i.e., Triscend, NaviGate, TriSol).

Data from randomized trials and registries have shown that most of the cases that require transcatheter tricuspid interventions are cases of functional (secondary) TR, mostly due to right ventricular dysfunction, tricuspid annular dilatation, and impaired leaflet coaptation [44, 45]. More than 90% of these patients have coexistence AF, requiring systemic anticoagulation regardless of the procedure [45, 46]. For the remaining patients, with no indication for systemic anticoagulation, similarly to other structural and valvular transcatheter interventions, DAPT consisting of 4 weeks of aspirin plus clopidogrel, followed by aspirin daily for life, is currently recommended.

In absence of dedicated, randomized studies, current practice include the extrapolation from recommendations with surgical bioprosthetic valves. For interventions in the tricuspid valve focusing the annulus or the leaflets, aggressive antithrombotic treatment seems not to be needed, rather than a short period of DAPT, until the device endothelialization. However, for cases of transcatheter tricuspid valve implantations, in the absence of an indication for antithrombotic therapy, OAC with VKAs for 6 months appears reasonable [47].

### 2.6 Patent Foramen Ovale, Atrial and Ventricular Septal Defects Transcatheter Closure

The PFO and the ASD represent the most common congenital heart diseases. Currently the indications for percutaneous closure include the prevention of recurrent paradoxical embolism in patients with diagnosis of PFO and important left-to-right shunt with signs of right ventricle overload and pulmonary vascular resistance lower than 5 Wood units in patients with secundum ASD [48].

PFO closure plays an important role for preventing recurrent stroke in patients with cryptogenic stroke in absence of any other intracardiac embolic source, or a stroke associated with major intracranial and extracranial vascular disorders [49]. Antithrombotic medication after the PFO closure is needed to avoid device thrombosis (2–3%) and embolization [50, 51, 52, 53]. Device thrombosis typically occurs on the metallic structures of the closure devices and develops early after implantation, within the first 4 weeks, caused by lack of endothelization in this initial period [50]. Of note, the endothelization of the device can continue up to five years post implantation, therefore early cessation of therapy may cause minor cerebrovascular events after PFO closure [52].

The optimal duration of antithrombotic therapy after PFO closure remains under debate. As of now, there are no definitive guidelines for medical management following PFO closure, except for the recommendation of antiplatelet therapy for secondary stroke prevention [54]. The most current recommendations for antithrombotic therapy after PFO closure depend on the specific indication for the procedure and individual patient factors and mainly extracted by the design of the pivotal REDUCE [55], RESPECT [56] and CLOSE [57] randomised trials that investigate the use of PFO occlusion devices, as compared with antiplatelet therapy.

In the REDUCE trial, 664 patients who had experienced a cryptogenic stroke were randomized in a 2:1 ratio to either undergo PFO closure using the Gore PFO occluder along with antiplatelet therapy or to receive antiplatelet therapy alone. The antiplatelet regimen included aspirin alone (75 to 325 mg once daily), a combination of aspirin (50 to 100 mg daily) and dipyridamole (225 to 400 mg daily), or clopidogrel (75 mg once daily). All patients remained at the prescribed antiplatelet therapy for a follow-up of 3.2 years. Notably, serious device-related adverse events were observed in 6 patients (1.4%) in the PFO closure group, and atrial fibrillation occurred in 29 patients (6.6%) following PFO closure [55].

In the RESPECT trial, 980 patients diagnosed with cryptogenic ischemic stroke were randomly assigned to either undergo PFO closure using the Amplatzer PFO occluder or receive medical therapy, with a follow-up duration of 5.9 years. Patients undergoing PFO closure were administered 81 to 325 mg of aspirin (ASA) along with clopidogrel 75 mg daily for one month, followed by ASA 81 mg for the subsequent five months. In the medical-therapy group, four regimens were permitted: ASA 81 mg daily, clopidogrel 75 mg daily, warfarin with a target INR of 2–3, and ASA plus dipyridamole (225 to 400 mg daily) [56].

In the CLOSE trial, 663 patients who had experienced cryptogenic stroke underwent randomization in a 1:1:1 ratio, with options for transcatheter PFO closure (utilizing various PFO occluders) combined with long-term antiplatelet therapy, antiplatelet therapy alone, or oral anticoagulation. Patients undergoing PFO closure were administered DAPT, consisting of 75 mg of ASA and 75 mg of clopidogrel, for a duration of 3 months, followed by SAPT for a follow-up period of 5.5 years. Among those assigned to oral anticoagulation, 93% received VKA, and 7% were on DOACs. In the antiplatelet therapy group, 87% were prescribed ASA 75 mg, 10% received clopidogrel 75 mg, and 3% were on a combination of ASA 75 mg and dipyridamole (225 to 400 mg daily) throughout the study period. Notably, the incidence of atrial fibrillation was higher in the PFO closure group compared to the antiplatelet-only group (4.6% vs. 0.9%, *p = *0.02) [57].

To conclude, in routine clinical practise, patients who undergo PFO closure due to a cryptogenic ischemic stroke, require DAPT with aspirin (81 to 100 mg) and clopidogrel 75 mg for a limited period, typically for 1–6 months, followed by ASA only (81 to 100 mg) for additional 4 to 8 months [55, 56, 57] (Table 4).

**Table 4.** **Summary of evidence regarding antithrombotic therapy following PFO/ASD/VSD closure**.

Antithrombotic therapy after PFO/ASD/VSD closure		
Amplatzer and Gore Occluders		Noble Stitch EL
High Bleeding risk and/or low thrombotic risk patients	Low bleeding risk patients and/or high thrombotic risk patients	Pretreatment with ASA 1 month
6–12 months ASA	1–6 months DAPT and then ASA lifelong	1–3 months DAPT. Then continue with ASA up to 12 months

Bleeding risk is considered elevated in elderly patients, in frailty, after history of GIH, elevated HAS-BLED score, anemia, thrombocytopenia, renal failure, hemorrhagic stroke etc. PFO, patent foramen ovale; ASA, aspirin; ASD, atral septal defect; VSD, ventricular septal defect; DAPT, dual antiplatelet therapy; GIH, gastro-intestinal hemorrhage.

Interestingly, PFO closure is associated with increased risk of new-onset AF [58]. Most device-associated AF incidences occurred early, were transient with no documented recurrence (76%), and only a minority of patients randomized to a device had stroke presumably caused by device-associated AF [59]. These short AF episodes most likely are related with peri-procedural factors as well with the type of the device, are transients with no documented relapse and are rarely causes of stroke [60]. Based on these characteristics a short-term (1–3 months) period of anticoagulation has been proposed [60].

A novel method of suture-mediated “deviceless” closure of PFO with the NobleStitch EL device has been tested in a small registry and was found feasible in most septal anatomies, providing an effective closure of PFO comparable to traditional devices with a good safety profile at medium-term follow-up. Patients were pretreated with and maintained on single antiplatelet therapy (preferably aspirin 100 mg) for approximately 1 month [61].

ASD is among the frequently observed congenital cardiac anomalies in adulthood. ASD is characterized by a flaw in the interatrial septum, enabling the direct passage of pulmonary venous return from the left atrium to the right atrium. Depending on the magnitude of the shunt, ASD can manifest with varying degrees of severity, ranging from an inconspicuous finding to a notable volume overload on the right side and the development of pulmonary arterial hypertension [62].

Device closure has become the first choice for secundum defect closure, when the procedure is feasible, based on morphology characteristics (diameter ≤38 mm and sufficient rim of 5 mm except towards the aorta) [63]. The type of the device and the site of the defect is like the PFO closure concept and since there are no dedicated studies for solely ASD closure, the current recommendations are based on the PFO trials. Therefore, after an ASD closure, antiplatelet therapy is required for at least 6 months (aspirin 75 mg o.d. minimum) [64].

Ventricular septal defects (VSDs) represent one the most prevalent forms of congenital heart disease, and surgical closure is widely acknowledged as the gold-standard treatment when deemed necessary [48]. Closure is typically recommended for VSDs leading to a Qp/Qs ratio exceeding 1.5 and resulting in volume overload in the left ventricle. In specific cases, percutaneous closure of VSD is considered a less invasive alternative to conventional open-heart surgery, particularly for membranous or muscular defects [65]. To ensure safe VSD closure using devices, it is essential to maintain an adequate distance (≥2 mm) from the aortic valves [66]. A variety of different devices have been used in the past for transcatheter VSD closure. Irrespectively of the device, recommendations regarding specific anticoagulation or antiplatelet therapy after device placement remain controversial [67]. Since there are no randomized trials available to assess the effectiveness of any of these treatment strategies against the other, all patients should receive a minimum of 74–100 mg aspirin for at least 6 months, similarly to the ASD closure procedure [68].

### 2.7 Left Atrial Appendage Occlusion

The majority of the thrombi causing stroke in patients with AF are formed in the LAA [69]. VKA and DOACs remain the gold standard therapy in patients with elevated thrombotic risk, assessed with the ${\mathrm{CHA}}_{2}$${\mathrm{DS}}_{2}$-VASc risk factors [70]. However, in patients with contraindication to receive antithrombotic medication or in an elevated bleeding risk, LAA occlusion (LAAO) remains a safe alternative [71].

The use of antithrombotic therapy in the immediate post-procedural period is required to minimize the risk of thrombus formation on the closure device. The incidence of device-related thrombus (DRT) has been reported to range between 4% and 17.6% [72, 73]. DRT is mostly related to technical factors (e.g., type of the device [74], uncovered pulmonary ridge, deep device implantation, peri-prosthetic leakage) or patient related factors (elevated ${\mathrm{CHA}}_{2}$${\mathrm{DS}}_{2}$-VASc risk score, ventricular dysfunction, advanced age) [75]. The most current recommendations for antithrombotic therapy after LAAO depend on the type of the device used for closure, individual patient factors, and the presence of other indications for anticoagulation or antiplatelet therapy (Table 5).

**Table 5.** **Summary of evidence regarding antithrombotic therapy following Left Atrial Appendage Closure**.

Antithrombotic therapy after Left Atrial Appendage Closure			
WATCHMAN		AMULET	
High Bleeding risk patients with contraindications to VKA	Low bleeding risk patients and/or elevated device-related thrombotic risk	High Bleeding risk patients with contraindications to VKA	Low bleeding risk patients
1 months DAPT (ASA with clopidogrel)	ASA + DOAC for 45 days. Then continue with DAPT for 6 months	1–3 months DAPT	3–6 months DAPT
Then continue with ASA for 12 months	Then continue with ASA for another 6 months	Then continue with ASA up to 12 months	Then continue with ASA alone

Device related thrombotic risk is considered elevated when is present history of stroke, heart failure, persistant AF, elevated ${\mathrm{CHA}}_{2}$${\mathrm{DS}}_{2}$-VASc score, enlarged left atrial appendage diameter, decreased left atrium velocities, important periprosthetic leak, and deep device implantation. Bleeding risk is considered elevated in elderly patients, in frailty, after history of GIH, elevated HAS-BLED score, anaemia, thrombocytopenia, renal failure, haemorrhagic stroke etc. AF, atrial fibrillation; ASA, aspirin; DAPT, dual antiplatelet therapy; DOAC, direct oral anticoagulation; VKA, vitamin k antagonists; GIH, gastro-intestinal hemorrhage.

The significant heterogeneity of the LAAO population, together with a wide variety of studies with different closure devices, different regimes and different outcomes make difficult a clear recommendation. Furthermore, antithrombotic therapy after LAAO has not been studied in a randomization fashion. Currently, the trend is to support the physician in the decision-making process for the choice of the suitable regimen post procedure, always taking under consideration the preference of the patient the bleeding and stroke risk, as well that the fact that all options (OAC, DOAC, DAPT) appear safe and effective.

For the cases where a WATCHMAN device has been used, the recommendations can be extracted from the landmark randomized clinical trials PROTECT-AF (Watchman Left Atrial Appendage System for Embolic Protection in Patients with Atrial Fibrillation) [76] and PREVAIL (Prospective Randomized Evaluation of the Watchman Left Atrial Appendage Closure Device in Patients With Atrial Fibrillation Versus Long Term Warfarin Therapy) [77]. These trials used a short period of VKA (45 days) with a longer course of ASA. At the day 45, the VKA was discontinued and Clopidogrel was used for up to 6 months after the procedure [76, 77].

Based on this data, the post implant drug regimen for patient’s prescribed short-term oral anticoagulation (OAK) with VKA is that patients should begin aspirin and warfarin with target and INR of 2.0 to 3.0 for at least 45 days post implant. After cessation of VKA the patient should remain on aspirin and begin clopidogrel until at least three months of elapse after implantation. Patients should remain on aspirin for at least 12 months after implantation [76, 77].

The more recent prospective registry EWOLUTION (Evaluating Real-Life Clinical Outcomes in Atrial Fibrillation Patients Receiving the Watchman Left Atrial Appendage Closure Technology) studied a higher risk population with elevated thrombotic risk (mean ${\mathrm{CHA}}_{2}$${\mathrm{DS}}_{2}$-VASc score 4.5 ± 1.6) and elevated bleeding risk, supporting the use of SAPT or DAPT alone (without OAC) after device implantation is safe and feasible [78, 79].

The post implant drug regimen for patients prescribed DAPT only, is that patients should begin clopidogrel and aspirin for at least 3 months post implantation. The EWOLUTION clinical study data establishing safety and effectiveness are based on demonstration of peri-device flow 5 mm as a measure of adequacy of LAA seal. If LAA seal is not demonstrated, the decision to discontinue clopidogrel is at physician discretion. Of note, patients should remain on aspirin at least 12 months after implantation. If thrombosis observed on the device use of anticoagulation again is at physician discretion [78].

Most of the studies that examine the antithrombotic treatment after the use of an AMPLATZER Cardiac Plug/Amulet device, found that ASA monotherapy after implantation [80, 81, 82] or DAPT with ASA and Clopidogrel for a period of 30 to 180 days [82] can be used without an increased risk of device-related thrombosis or stroke. A recent observational study, a comparison of SAPT versus DAPT in patients who underwent LAAO, for patient treated either with WATCHMAN or AMULET, showed that post-procedural use of SAPT instead of DAPT was associated with reduction of bleeding complications, with no significant increase in the risk of thrombotic events [83]. In the absence of significant peri-device flow or device-related thrombus (DRT), short-term DAPT for six weeks followed by single antiplatelet therapy appears to be a viable alternative for patients after LAAO [84]. In summary, for patients at a high risk of bleeding, adopting a strategy of 1- to 6-month DAPT involving low-dose aspirin and clopidogrel (preferably continued until sufficient sealing of the left atrial appendix) seems appropriate, followed by an extended period of single antiplatelet therapy (Table 5).

### 2.8 Challenges and Future Directions

Despite the growing use of structural heart disease interventions, there is a relative lack of robust randomized clinical trials and high-quality evidence specifically addressing antithrombotic therapy in these settings. Many recommendations are based on expert consensus or extrapolation from studies focused on other cardiovascular interventions.

Well-designed randomized controlled trials (RCTs) comparing different antithrombotic regimens, durations, and intensities of therapy are needed to provide stronger evidence for guiding treatment decisions. Furthermore, subgroup analyses within existing trials or registry-based studies can provide valuable insights into specific patient populations and procedural nuances. Collaboration among multiple centers or international consortia can help facilitate larger-scale studies to address these gaps.

One of these gaps is related to the effectiveness of clopidogrel, a widely used antiplatelet medication in structural interventions. The effectiveness of clopidogrel can be hindered by certain limitations, notably the heightened ischemic risk observed in individuals with high platelet reactivity and genetic variations impacting the CYP2C19 enzyme [85]. Clopidogrel is characterised by high interindividual variability in platelet inhibition and a large proportion of patients are not-responders. This fact, in the field of coronary interventions, underlined the need for more potent and consistent platelet inhibition that was covered with novel generation P2Y12 inhibitors [86]. However, in the field of structural heart disease interventions, clopidogrel was ever since the studied regimen.

Platelet reactivity can be measured with the commercially available VerifyNow assay (Accriva Diagnostics, San Diego, CA, USA). The recent Assessment of platelet REACtivity after Transcatheter Aortic Valve Implantation (REAC-TAVI) trial enrolled patients with aortic stenosis (AS) undergoing TAVI pre-treated with aspirin and clopidogrel, aimed to compare the efficacy of clopidogrel and ticagrelor in suppressing high platelet reactivity (HPR) after TAVI [87]. The study showed that HPR to clopidogrel is present in a considerable number of patients with AS undergoing TAVI. However, in this elderly population with a high risk of bleeding the high level of platelet inhibition achieved with a potent antiplatelet agent might considered as a drawback. The ongoing, TICTAVI study (NCT02817789), which will investigate further the impact of ticagrelor monotherapy for 30 days after valve implantation vs. DAPT with clopidogrel and the soluble salt of aspirin lysine acetylsalicylate. Anticoagulation therapy has seen significant advancements in the past decade, primarily attributed to the development of FXa inhibitors [88]. However, existing anticoagulants, both direct and indirect, lack specificity in distinguishing between pathological coagulation (thrombosis) and physiological coagulation (haemostasis). Despite their clinical effectiveness, these agents often lead to substantial bleeding complications, particularly in specific patient groups such as those with chronic kidney disease [89]. Novel anticoagulants may address these challenges by targeting coagulation proteins of the intrinsic and contact activation pathways, such as factor XIa. Focusing on FXIa could enhance anticoagulant effects while minimizing bleeding risks, given its predominant role in pathological blood clotting (thrombosis) and a lesser role in physiological blood clotting (haemostasis) [90].

Innovative approaches to antithrombotic treatment, such as targeting factor XI/XIa or XII/XIIa through small-molecule inhibitors, antibodies, or antisense oligonucleotides, are actively under development. These emerging strategies hold promise for effectively inhibiting the contact activation pathway on artificial devices, presenting novel and compelling avenues for intervention [91, 92].

Thromboelastography (TEG or ClotPro) and rotational thromboelastometry (ROTEM) are advanced hemostatic monitoring techniques used to assess intrinsic thrombotic propensity or bleeding propensity in heart interventions [93]. These point-of-care tests provide dynamic information on the entire coagulation process, offering insights into clot formation, strength, and breakdown. By evaluating these parameters, clinicians can tailor anticoagulation and antiplatelet strategies more precisely during heart interventions, optimizing the delicate balance between preventing thrombosis and minimizing bleeding risks.

The management of patients undergoing structural cardiac interventions presents an additional challenge due to gender-related differences. Gender-related variances in the management of patients undergoing structural heart disease interventions have gained increasing attention in cardiovascular care. While the prevalence of structural heart disease and valvular conditions may vary between genders, research has also explored potential variations in the diagnosis, treatment, and outcomes based on gender [94]. Given the perception of women as a potentially more delicate group susceptible to bleeding, they often receive less aggressive treatment in clinical settings. Nevertheless, multiple studies have highlighted comparable thrombotic risks between genders, with a tendency towards increased bleeding risk in females [95]. This inclination is often influenced by factors such as advanced age, lower body weight, higher comorbidity rates, and potential overuse of antithrombotic medications. Of note, the benefits of aspirin for cardiovascular risk, in both primary and secondary prevention, has shown no significant gender-related differences [96]. Similarly, there is no indication of significant gender-related differences in the context of anticoagulant drugs [94].

Optimal antithrombotic therapy following structural heart disease interventions requires an individualized approach. Patient-specific factors such as age, comorbidities, bleeding risk, thrombotic risk, and procedural characteristics need to be carefully considered. Currently, there is limited guidance on how to balance the risks of thrombosis and bleeding in specific patient subgroups. Developing risk stratification models and decision-making tools can assist in tailoring therapy to individual patients.

Besides, achieving the delicate balance between preventing thrombotic events and minimizing bleeding complications is a major challenge in antithrombotic therapy after these interventions. There is a need to define optimal duration and intensity of therapy, as prolonged use of antithrombotic agents may increase bleeding risk without substantial benefit, while premature discontinuation may increase the risk of thrombosis. Patients should stratify based on thrombotic and bleeding risk, along with refining risk prediction models, can help guide treatment decisions and strike an appropriate balance.

In the pursuit of tailoring antithrombotic treatment to individuals undergoing cardiac interventions, significant efforts have been dedicated in the past decade to enhance the identification of patients with an elevated risk of bleeding complications. Numerous risk algorithms and scores are utilized to assess the significance of specific clinical, laboratory, and technical factors [97]. While most of these risk scores have been designed and validated for identifying bleeding risk in patients’ post-percutaneous coronary intervention, their validation within the realm of structural heart disease interventions is lacking. Among them, the HAS-BLED score which was initially developed to assess bleeding risk in patients with atrial fibrillation who are receiving anticoagulant therapy, has been applied to other settings, including structural cardiac interventions [98].

Frailty is a prevalent condition among those undergoing TAVI and other valvular heart disease interventions and can influence their overall management. Many studies indicate that the risks of both short and long-term mortality, as well as bleeding complications, rise with increasing degrees of frailty [99, 100, 101]. Consequently, there is a suggestion to incorporate preoperative screening tools, including both geriatric and nongeriatric scales such as the Hospital Frailty Risk Score and the Clinical Frailty Scale, to enhance the optimization of TAVI care pathways and refine antithrombotic strategies associated with the procedure [102].

More recent, the Valve Academic Research Consortium criteria provided standardized definitions for various clinical events related to valvular heart disease interventions. The VARC criteria were developed to facilitate consistent reporting of clinical outcomes mainly in TAVI studies and trials [103, 104]. Especially for bleeding events, the VARC-2 criteria define three levels of severity: minor, major, and life-threatening or disabling bleeding [103]. The most recent updated VARC-3 criteria have been modified into a more descriptive classification scheme: type 1 (minor), type 2 (major), type 3 (life-threatening), and type 4 (leading to death) bleeding [104]. When evaluating bleeding risk in the context of TAVI or other structural heart disease procedures, clinicians may use these VARC definitions in conjunction with other bleeding risk assessment tools and considerations, such as patient history, laboratory assessments, and procedural factors. The goal is to tailor the assessment and management to the specific needs of each patient undergoing structural interventions. It’s important to note that the applicability of specific scores may vary depending on the context and the population being studied. Additionally, individual patient characteristics and local practices may influence the choice of risk assessment tools.

Addressing these challenges and knowledge gaps requires collaborative efforts among clinicians, researchers, industry, and regulatory bodies. Conducting well-designed clinical trials, generating real-world evidence, and leveraging emerging technologies will help bridge the gaps in knowledge and provide evidence-based guidelines for antithrombotic therapy following structural and valvular heart disease interventions.

## 3. Summary and Conclusion

In conclusion, antithrombotic therapy following structural heart disease interventions is a rapidly evolving field. Although current recommendations provide general guidance, personalized approaches based on individual patient factors are essential. Future research should focus on generating high-quality evidence, developing tailored strategies, and exploring novel agents and technologies to further enhance patient outcomes in this exciting and evolving field of interventional cardiology.

## Abbreviations

AF, Atrial Fibrillation; ASA, Aspirin; ASD, Atrial Septal Defect; AVR, Aortic Valve Replacement; DAPT, Dual Antiplatelet Therapy; DOACs, Direct Oral Anticoagulants; HPR, High Platelet Reactivity; INR, International Normalized Ratio; LAA, Left Atrial Appendage; LAAO, Left Atrial Appendage Occlusion; MR, Mitral Regurgitation; MVR, Mitral Valve replacement; OAC, Oral Anticoagulation; PFO, Patent Foramen Ovale; RCT, Randomized Control Trials; SAS, Severe Aortic Stenosis; SAPT, Single Antiplatelet Therapy; TAVI, Transcatheter Aortic Valve Implantation; TEER, Transcatheter Edge to Edge Repair; TMVR, Transcatheter Mitral Valve Replacement; TR, Tricuspid Regurgitation; VARC, Valve Academic Research Consortium; ViMAC, Valve in MAC; ViR, Valve in Ring; ViV, Valve in Valve; VKA, Vitamin K antagonists; VSD, Ventricular Septal Defect.
